# Regulating Polyamine Metabolism by miRNAs in Diabetic Cardiomyopathy

**DOI:** 10.1007/s11892-021-01429-w

**Published:** 2021-12-13

**Authors:** Tyler N. Kambis, Hadassha M. N. Tofilau, Flobater I. Gawargi, Surabhi Chandra, Paras K. Mishra

**Affiliations:** 1 Department of Cellular and Integrative Physiology, University of Nebraska Medical Center, Omaha, NE 68198 USA; 2 Department of Biology, University of Nebraska-Kearney, Kearney, NE 68845 USA

**Keywords:** Diabetes, Heart, Insulin, Ketone body, T1DM, T2DM, Urea cycle, Methionine cycle

## Abstract

**Purpose of Review:**

Insulin is at the heart of diabetes mellitus (DM). DM alters cardiac metabolism causing cardiomyopathy, ultimately leading to heart failure. Polyamines, organic compounds synthesized by cardiomyocytes, have an insulin-like activity and effect on glucose metabolism, making them metabolites of interest in the DM heart. This review sheds light on the disrupted microRNA network in the DM heart in relation to developing novel therapeutics targeting polyamine biosynthesis to prevent/mitigate diabetic cardiomyopathy.

**Recent Findings:**

Polyamines prevent DM-induced upregulation of glucose and ketone body levels similar to insulin. Polyamines also enhance mitochondrial respiration and thereby regulate all major metabolic pathways. Non-coding microRNAs regulate a majority of the biological pathways in our body by modulating gene expression via mRNA degradation or translational repression. However, the role of miRNA in polyamine biosynthesis in the DM heart remains unclear.

**Summary:**

This review discusses the regulation of polyamine synthesis and metabolism, and its impact on cardiac metabolism and circulating levels of glucose, insulin, and ketone bodies. We provide insights on potential roles of polyamines in diabetic cardiomyopathy and putative miRNAs that could regulate polyamine biosynthesis in the DM heart. Future studies will unravel the regulatory roles these miRNAs play in polyamine biosynthesis and will open new doors in the prevention/treatment of adverse cardiac remodeling in diabetic cardiomyopathy.

**Supplementary Information:**

The online version contains supplementary material available at 10.1007/s11892-021-01429-w.

## Introduction

Impaired glucose metabolism due to insulin deficiency (T1DM) or insulin resistance (T2DM) causes cardiac metabolic remodeling in diabetes mellitus (DM) [1]. The DM heart develops a cardiac muscle disorder called diabetic cardiomyopathy (DMCM), which leads to heart failure [2•]. DMCM causes steady progressive remodeling in the heart, exhibiting different features at different stages: early stage — increased oxidative stress and inflammation; middle stage — increased cell death, fibrosis, and diastolic dysfunction; and late stage — diastolic and systolic dysfunction [3]. At the crossroad of these pathologies is metabolic dysregulation, a significant part of which is consists of impaired polyamine biosynthesis.

Polyamines are charged organic compounds with insulin-like actions that affect the metabolism of both lipids and glucose [4•, 5, 6]. The rate-limiting enzyme of polyamine biosynthesis is ornithine decarboxylase (ODC). In streptozotocin-induced T1DM rats, levels of ODC are markedly lower in the heart, liver, and skeletal muscles but retain normal levels in insulin-treated T1DM rats [7]. Thus, polyamine biosynthesis may be regulated by insulin. High glucose decreases levels of the polyamine spermine in primary neonatal rat cardiomyocytes, while simultaneously impairing mitochondrial function by reducing mitochondrial membrane potential and increasing ATP leakage. Supplementation of spermine, another polyamine, in these cardiomyocytes prevented high glucose-induced loss of mitochondrial membrane potential and improved ATP levels [8]. Therefore, polyamines, such as spermine, could be important in preserving mitochondrial energetics in a high glucose environment. Moreover, supplementation of spermine improves glucose uptake, reduces myocardial cell death and oxidative stress, and attenuates cardiac dysfunction in the streptozotocin-induced T1DM heart [9]. In alloxan-induced DM rats, separate treatments with insulin and polyamines were compared in their ability to maintain serum levels of glucose and beta-hydroxybutyrate — a ketone body associated with mitochondrial damage and cardiotoxicity. Polyamines reduced the levels of glucose and beta-hydroxybutyrate comparable to that of insulin, suggesting that polyamines and insulin signaling work in parallel for controlling the circulating levels of glucose and ketone bodies in DM [10••]. These findings suggest that polyamines have critical protective roles in myocardial metabolism and adverse cardiac remodeling in DM. However, it remains unclear as to how the biosynthesis of polyamines is regulated in the DM heart.

The majority of biological functions are regulated by microRNAs-tiny (18–25 nucleotides long), endogenous, non-coding RNAs that fine tune gene expression by mRNA degradation or translational repression [11•, 12]. A single microRNA (miRNA) can regulate several genes in a biological pathway and thus could have therapeutic potential for cardiovascular diseases [13•]. Levels of many miRNAs that regulate metabolism are altered in the DM heart [14–16]. How miRNA regulates cardiac polyamine biosynthesis in the DM heart is unknown.

Here, we searched for potential miRNAs that are present in the heart and have predicted targets in the polyamine biosynthesis pathway. We also elaborated the potential roles of polyamines in cardiac metabolism and maintaining ketone body levels — a crucial step in metabolism to control ketoacidosis in DM. This review sets a platform to explore the regulatory roles of polyamines in mitochondrial dysfunction and cardiac metabolic derangement during DMCM and investigate the potential of miRNAs to regulate levels of polyamines in the DM heart.

## Regulation of Polyamine Levels

Polyamines are organic compounds with two or more amino groups that render them a high positive charge, thus also gaining the term polycations [17•]. They were first discovered in seminal fluids [18•]. One important function of polyamines is the translational regulation of genes [19, 20]. The uptake, biosynthesis, and catabolism of polyamines are briefly described below:

### Polyamine Uptake

Polyamines from the diet are transported through specific uptake mechanisms; however, there is a lack of unifying theory behind this [21]. Some transporters that have been suggested so far include the solute carrier SLC3A2, with selectivity towards putrescine polyamine [22, 23], and the vesicular solute carrier SLC18B, with selectivity towards spermidine and spermine polyamines [24]. An alternate transport pathway utilizes endocytic uptake of polyamines through glypicans, which are heparan sulfate groups within plasma membrane proteins [25]. The lysosomal/endosomal transporter ATP13A3 has also been suggested for the movement of putrescine, spermine, and spermidine into the cytosol [26•].

### Polyamine Biosynthesis

**Fig. 1:**
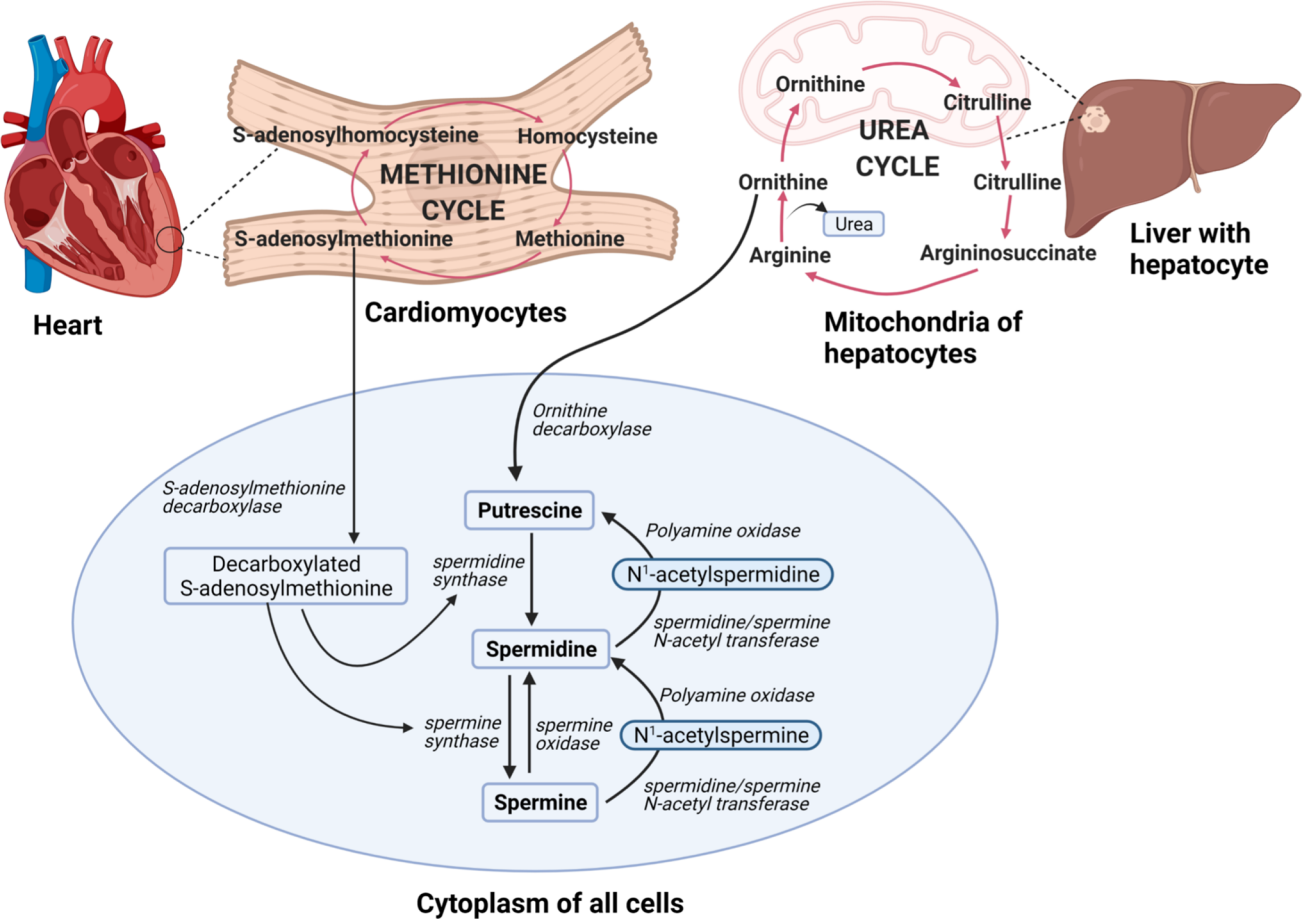
The intracellular polyamine metabolic pathway. The primary polyamine metabolic pathway occurs in the cytoplasm of all cells and involves the formation of polyamines (putrescine, spermidine, and spermine) from ornithine through ornithine decarboxylase enzyme. The intracellular source of ornithine is from the urea cycle in the liver. S-adenosylmethionine metabolism is a parallel pathway that contributes to synthesis of polyamines. This is a part of the methionine cycle which occurs in the cytoplasm of all cells, including cardiomyocytes. Figure created using BioRender

While the primary exogenous source of mammalian polyamines is through nutrition, most of the intracellular polyamine pool is regulated through cellular synthesis or synthesis by the gut microbiome [27, 28]. Gut microbiome-triggered polyamine production is predominantly responsible for colorectal cancer [29]. Polyamines are synthesized from amino acids — arginine, ornithine, and methionine — and rely on unique enzymes to harness each amino acid pool separately [30, 31]. Arginase converts arginine to ornithine and exists in two isoforms-arginase 1 (cytoplasmic) and arginase 2 (mitochondrial) [32]. Although predominantly found in the liver, arginase 1 is also present in endothelium, smooth muscle, and neuronal cells [33–36]. Ornithine is metabolized by ODC to produce the first polyamine, putrescine. ODC is the rate-limiting enzyme of the polyamine biosynthesis pathway, and as such is tightly regulated at the transcriptional and post-transcriptional levels [37, 38]. Spermidine synthase biosynthesizes the second polyamine, spermidine, from putrescine. Spermidine is converted into the final polyamine, spermine, via the enzyme spermine synthase (Fig. 1).

Another pathway for polyamine biosynthesis is the methionine cycle, which is present in most cells [39]. Methionine is shuttled as S-adenosyl methionine via S-adenosylmethionine synthase (SAMS). This is further metabolized through S-adenosylmethionine decarboxylase to decarboxylated S-adenosylmethionine, which can replenish polyamines pool through spermidine synthase (for spermidine) and spermine synthase (for spermine) (Fig. 1). In several pathological conditions, such as hepatic carcinoma, lung cancer, and prostate cancer, the levels of S-adenosylmethionine are elevated [40, 41, 42•].

### Polyamine Catabolism

While non-reversible, it is possible to recycle spermine and spermidine through the action of N-acetyltransferase intermediates [43, 44]. Spermine can be converted to spermidine through the action of spermine oxidase (SMOX). Alternatively, spermine or spermidine can be converted to lower polyamines through a two-step reaction involving spermidine/spermine N^1^-acetyltransferase (SAT1) and acetylpolyamine oxidase. This is an important step in polyamine catabolism as SAT1 is the primary driving force for maintaining intracellular polyamine pools. Upregulation of SAT1 drives spermine catabolism, which activates the biosynthesis of spermidine. Spermine is also catabolized by spermine oxidase to form spermidine, thus maintaining polyamine concentrations [42•] (Fig. 1). Oxidative deamination of polyamines by Cu^2+^-dependent diamine oxidase plays an important role in the terminal catabolism of polyamines, which has been discussed at length in a previous review [43].

## Protective Roles of Polyamines in Diabetes Mellitus

Polyamine metabolism affects DM by improving insulin action and preserving pancreas function. Polyamine biosynthesis is stimulated by glucose uptake in islet cells [44, 45]. Increases in the level of insulin due to elevated glucose levels is paralleled by an increase in polyamine synthesis that inhibits pro-insulin transcription [46]. It is known that polyamines, specifically spermidine and spermine, regulate insulin signaling via interaction with insulin-like-growth factor-1 and by increasing the transcription of the proinsulin gene [46, 47]. SAT1 knockout mice exhibit insulin resistance upon aging, while SAT1 overexpressing mice demonstrate improved glucose tolerance [48]. Increasing catabolism by depletion of internal polyamine pools also protects against the development of hyperglycemia by preserving pancreatic beta cell survival in chemically-induced DM in preclinical models [49]. In addition to this, deoxyhypusine synthase utilizes spermine to induce adaptive pancreatic beta cell proliferation under insulin resistant conditions [50]. Furthermore, polyamine oxidase is increased in the serum of diabetic children, providing a potential role of polyamine oxidase in increased levels of circulatory oxidative stress [51•]. The serum levels of putrescine are elevated in patients with T2DM, corroborating that polyamine metabolism is dysregulated in DM [52••].

## Evidence of Polyamines Involvement in Cardiomyopathy

Polyamines are involved in the cardiac development of rats and mice [53•, 54••]. Polyamine dysregulation is linked to cardiovascular diseases caused by ischemia/reperfusion (IR) injuries, suggesting a potential of polyamines in adverse cardiac remodeling [54••]. However, a direct link between polyamines and cardiomyopathy has not been established. A distinct hallmark in ischemic cardiomyopathy is increased expression of N^8^-acetylspermidine (N8AS) [55•]. N8AS is formed from spermidine by the enzyme SAT1 and converted into spermidine by histone deacetylase [55•]. Overexpression of polyamine metabolic enzymes such as ODC and SAT1 has been linked to ischemic cardiomyopathy, and polyamines like N8AS serve as a potential biomarker for ischemic cardiomyopathy. Other polyamines such as spermidine and spermine are downregulated in cardiomyopathy, possibly due to consumption of spermine in preventing myocardial apoptosis [56•, 57]. In cirrhotic cardiomyopathy, an increase in spermidine offers a protective effect by reducing inflammation and increasing antioxidant enzymes [58]. Other studies on rats have shown that spermidine provides protection against heart injury by preventing oxidative stress [59•]. These findings support the potential cardioprotective roles of polyamines in cardiovascular diseases and cardiomyopathy.

## Polyamines and Cardiac Metabolism

The pathway of polyamine biosynthesis is tied upstream to the TCA cycle, linking it to all major metabolic pathways. In patients with ischemic cardiomyopathy, plasma levels of N8AS correlate with an increase in metabolites in the carnitine shuttle and an oversaturation of the fatty acid β‐oxidation pathway [55•]. RNA sequencing performed in rat hearts revealed that spermine and spermidine treatment prevented cardiac age-related deterioration by restoring pathways associated with fatty acid metabolism [60]. In neonatal rat cardiomyocytes, spermidine treatment restored hypoxia-impaired state 3 and state 4 mitochondrial respiration [59•]. Spermidine feeding also enhanced mitochondrial respiration in aged mice and hypertensive rats, leading to improved cardiomyocyte composition and cardiac function [61••]. Heart failure, which typically decreases fatty acid metabolism, is associated with a significant increase in levels of putrescine, spermine, ornithine, and arginine along with a decrease in levels of several lipid intermediates in mouse hearts [62]. Mass spectrophotometric analysis conducted in 12/15LOX^−/−^ mice undergoing ischemic heart failure indicated metabolic reprograming via an upregulation in levels of the amino acids arginine, ornithine, methionine, and the polyamines spermidine and spermine, suggesting a preference for amino acid entry directly into polyamine biosynthesis over the Krebs cycle in failing hearts with the impaired presence of fatty acid-metabolizing enzymes [63]. Ketogenesis, upregulated during fatty acid metabolism over-saturation, was found to be transiently activated, while arginine, methionine, and S-adenosylmethionine were found to be consistently decreased across postnatal mouse heart development [64]. Interestingly, treatment with L-arginine, spermidine, spermine, and putrescine in pregnant diabetic mice significantly lowered circulating level of beta-hydroxybutyrate (a ketone body), suggesting a ketogenic regulatory role of polyamines [10••].

## Potential Roles of Polyamines in Diabetic Cardiomyopathy

DM induces DMCM, leading to an increased risk of heart failure. T2DM increases the risk of heart failure in men 2.4-fold and in women 5.1-fold [65], while T1DM increases the risk of heart failure fourfold independent of sex [66]. While there is limited clinical data examining polyamines in DMCM, there are several preclinical studies focusing on determining the role of polyamine biosynthesis in the DM heart. Exogenous spermine attenuates DMCM by suppressing ROS-p53-mediated downregulation of calcium-sensitive receptors and inhibiting Wnt/β-catenin signaling [9, 67•]. Downregulated calcium-sensitive receptor expression has been tied to DMCM, supporting the concept that downregulated spermine could contribute to DMCM [68]. Furthermore, ODC activity is 77% lower in the hearts of T1DM rats and is rescued by treatment via insulin [7, 69•]. Cardiac insulin binding is improved by treatment with spermine and spermidine via increased cardiac insulin receptor tyrosine kinase activity, corroborating a role of impaired polyamine biosynthesis in T2DM-induced insulin resistance [70]. Additional studies have shown that high glucose was able to induce fibrosis in rat cardiac fibroblasts through increased arginase activity. Upregulation of arginase activity is attributed to increased levels of intracellular ROS in hyperglycemic H9C2 cardiac myoblasts, while inhibition of arginase via remote ischemic preconditioning significantly preserved myocardial infarct size in T1DM, suggesting a potential maladaptive mechanism to the over activation of upstream enzymes in the polyamine biosynthesis pathway in DMCM [71, 72]. Furthermore, spermidine consumption has been shown to worsen myocardial lipid deposition, a precursor of DMCM, in high fat-diet-induced obese male mice [73].

A summary of expression levels of various polyamines and polyamine metabolic enzymes that have potential to contribute to DMCM is presented in Table 1. One of the challenges with linking polyamines to DMCM is the change in expression levels of polyamines over the course of disease progression. An increase or decrease in polyamine levels may either increase or decrease injury in a dose-dependent fashion in ischemia–reperfusion injury [74•]. There is a lack of human studies determining the role of polyamines in cardiac dysfunction and the regulation of polyamine biosynthesis and metabolism.

**Table 1:** Polyamine markers in DMCM and non-DMCM**.** ↑/↓ indicates the upregulation/downregulation of the polyamine marker determined relevant to the specific model of cardiomyopathy. (*ODC*, Ornithine Decarboxylase; *SAT1*, spermidine/spermine N1-acetyltransferase; *N8AS*, N^8^-acetylspermidine)

Polyamine marker	Expression	Cardiomyopathy	PMID
ODC	↑	Dilated	[54••]
ODC	↓	Diabetic	[69•]
SAT1	↑	Dilated	[54••]
SAT1	↓	Diabetic	[6]
Spermine	↓	Dilated^1^, diabetic^2^	[54••]^1^, [67•]^2^
N8AS	↑	Dilated	[55•]
Spermidine	↓	Cirrhotic	[58]
Spermidine	↑	Diabetic	[6, 73]
Putrescine	↓	Diabetic	[73]
Arginine	↑	Diabetic	[72]

Superscripts correspond to respective citations

## Potential miRNAs Involved in Regulation of Cardiac Polyamines in Diabetic Cardiomyopathy

DMCM is characterized by a dysregulation of the miRNA regulatory network. Out of the total cataloged 2000 mature miRNAs, approximately 400 are expressed in the human heart [75]. Of these 400 miRNAs, the expression of approximately 2 dozen is changed in the human heart (Table 2). Of these miRNAs, miR-1, miR-34a, miR-34b, miR-34c, miR-199a, miR-199b, miR-204, miR-208a, miR-210, miR-216a, miR-223, miR-320, miR-372, and miR-485 are upregulated while miR-9, miR-23b, miR-29b, miR-126, miR-132, miR-133a, miR-181a, miR-182, miR-214, and miR-221 are downregulated. Not all miRNA expression follows the same change in trend between T1DM and T2DM hearts. miR-21, miR-34a, miR-141, and miR-499 have opposite changes in rodent cardiac tissue expression between T1DM and T2DM hearts. Furthermore, in vitro studies have allowed a better understanding of the effects of dysregulated miRNAs on DM-induced cardiac remodeling pathways. Many cardiac miRNAs are involved in regulating cell death, with miR-1, miR-19, miR-34a, miR-195, miR-320, and miR-483 acting as pro-apoptotic whereas miR-30d as pro-pyroptotic. miR-26a, miR-26b, miR-30c, miR-144, and miR-181a are anti-apoptotic, while miR-9 is anti-pyroptotic. miR-195 induces oxidative stress, while miR-21, miR-144, and miR-373 each prevent the generation of oxidative stress. miR-133a, miR-150, miR-181a, and miR-373 are anti-hypertrophic, while miR-195 and miR-451 are pro-hypertrophic. Lastly, miR-29 and miR-141 promote DM-induced cardiac fibrosis whereas both miR-30c and miR-200b work to restore metabolism in the diabetic myocardium (Table 3).

**Table 2:** Human miR expression in DMCM. A search of the literature utilizing PubMed was conducted to find published microRNA expression in diabetic human heart tissue samples. ↑/↓ indicates the upregulation/downregulation of the microRNA. Only research articles from the last 10 years were considered. In total, 24 microRNAs were selected that have different expressions in diabetic human heart samples compared to the normal human heart

miRNA	Expression	PMID
miR-1	↑	[87]
miR-9	↓	[89]
miR-23b	↓	[90]
miR-29b	↓	[91]
miR-34a	↑	[79, 92]
miR-34b	↑	[80]
miR-34c	↑	[80]
miR-126	↓	[93, 94]
miR-132	↓	[94]
miR-133a	↓	[80, 95]
miR-181a	↓	[96]
miR-182	↓	[80]
miR-199a	↑	[80]
miR-199b	↑	[80]
miR-204	↑	[80]
miR-208a	↑	[97]
miR-210	↑	[80]
miR-214	↓	[80]
miR-216a	↑	[80]
miR-221	↓	[80]
miR-223	↑	[98]
miR-320	↑	[99]
miR-372	↑	[80]
miR-485-5p	↑	[80]

**Table 3:** In vitro miRNA expression in DMCM. A PubMed search was conducted to find the established microRNA expression in cardiomyocytes. ↑/↓ indicates the upregulation/downregulation of the microRNA determined in diabetic heart models. Only research articles from the last 10 years were considered. In total, 22 microRNAs were found to have differential expressions in treated cardiomyocytes (glucose, fatty acid), compared to normal conditions. microRNA target, pathway, and treatment was specified based on the research findings and data. H9C2 is a cardiomyoblasts cell line derived from ventricle embryonic rat heart tissue. HL-1 is a cardiomyocyte cell line derived from left atrial mouse heart tissue. AC-16 is a human cardiomyocyte cell line derived from adult ventricular heart tissue

miRNA	Levels	Pathway	Model	PMID
miR-1	↑	Apoptosis	Rat cardiomyocytes^1,2^, H9C2^1^, HL-1^2^	[100]^1^, [101]^2^
miR-9	↓	Pyroptosis	H9C2	[89]
miR-19	↑	Apoptosis	H9C2	[102]
miR-21	↓	Oxidative stress	Rat cardiomyocytes	[103]
miR-26a	↓	Apoptosis/fibrosis	Mouse cardiomyocytes	[104]
miR-26b	↓	Apoptosis/fibrosis	Mouse cardiomyocytes	[104]
miR-29	↑	Cardiac fibrosis	Mouse cardiac fibroblast	[105]
miR-30c	↓	Apoptosis^1^, metabolism^2^	Rat cardiomyocytes^1^, H9C2^2^	[96]^1^, [106]^2^
miR-30d	↑	Pyroptosis	Rat cardiomyocytes	[107]
miR-34a	↑	Apoptosis	AC-16^1^, CPCs^1^, H9C2^2^	[79]^1^, [108]^2^
miR-133a	↓	Hypertrophy	Rat cardiomyocytes	[109]
miR-141	↑	Cardiac fibrosis	Mouse cardiac fibroblast	[110]
miR-144	↓	Apoptosis, oxidative stress	HL-1	[111]
miR-150	↓	Hypertrophy	Rat cardiomyocytes	[112]
miR-181a	↓	Apoptosis, hypertrophy	H9C2	[96]
miR-195	↑	Oxidative stress, apoptosis, hypertrophy	Rat and mouse cardiomyocytes	[113]
miR-200b	↓	Metabolism	H9C2, Rat cardiomyocyte	[84]
miR-200c	↑	DUSP-1, MAPK signaling	Rat cardiomyocyte	[85]
miRNA-320	↑	Apoptosis	Rat cardiomyocytes	[114]
miR-373	↓	Hypertrophy, oxidative stress	Rat cardiomyocytes	[115]
miR-451	↑	Hypertrophy	Rat cardiomyocytes	[116]
miR-483	↑	Apoptosis	H9C2	[117]

Superscripts correspond to respective citations

## Predicted miRNA and Their Targets in Polyamine Biosynthesis Pathway in Diabetic Cardiomyopathy

While there are no studies showing a direct relationship between miRNAs and polyamine biosynthesis in DMCM, there is some data supporting roles of those miRNAs that are deregulated during DMCM in the regulation of polyamine biosynthesis in other diseases. miR-34a and miR-34b, which are upregulated in DMCM, target methionine adenosyl transferase 2A (MAT2A). S-adenosylmethionine (SAM) and methylthioadenosine (MTA) upregulate miR-34a and miR-34b in colorectal cancer cells [76]. Cellular levels of polyamines in intestinal epithelial cells decrease miR-29b via decreasing JunD-expression levels, mimicking miR-29b’s expression in the DM heart [77]. S-adenosyl-L-methionine (AdoMet) upregulates miR-34c in triple negative breast cancer cell lines while inducing a pro-apoptotic cell death effect. miR-34c is also increased in the DM heart, while miR-34a has been established to be both upregulated in the DM heart and be pro-apoptotic [78, 79]. Decreased levels of miR-199a-5p are also correlated with increased aggressive pituitary tumor growth via targeted inhibition of SAT1. miR-199a is also increased in the DM heart [80]. miR-210, which is both pro-cancerous and overexpressed in DMCM, significantly increased levels of all metabolites in HEK293 cells, including putrescine and spermidine [80, 81]. miR-372, another oncogenic miRNA upregulated during DMCM, was the first established miRNA to be inhibited via polyamine derivatives [80, 82]. Furthermore, knockdown of miR-485-3p in Hep3B and HepG2 liver cell lines induced methionine adenosyl-transferase 1A (MAT1A) expression [83]. miR-200a targets Keap1 to allow NRF2 binding to SAT1 in non-small-cell lung carcinoma cell lines. While miR-200a expression is not changed in the DM heart, miR-200b and miR-200c expressions are different [84, 85]. This remains relevant, as the miR-200a and miR-200b/c seed sequence varies by only a single nucleotide [86]. In addition to these potential miRNA regulators of polyamine metabolism, there are extensive mRNA gene-interaction analysis conducted utilizing microarray and immunoprecipitation showing interactions between miRNAs disrupted during DMCM and polyamine biosynthesis enzymes (Table 4).

**Table 4:** Predicted cardiac miR polyamine targets. Polyamine biosynthesis enzymes potentially targeted by miRNAs dysregulated during DMCM. Table generated utilizing Tarbase v.8. miRNA-Gene interaction predicted via immunoprecipitation/microarray

Target	miRNA	Tissue	Model	PMID
ODC	hsa-miR-133a-3p	Cervix, pancreas	HELA, PBC, BCBL	[118–120]
ODC	hsa-miR-1-3p	Cervix, kidney	HELA, 293S	[121, 122]
ODC	hsa-miR-133b	Bone marrow	BCBL	[120]
ODC	hsa-miR-126-3p	Bone marrow	AML8227	[123]
ODC	hsa-miR-34a-5p	Bone marrow	K562	[124]
ODC	hsa-miR-126-5p	Kidney, umbilical vein	HEK293, HUVEC	[125–127]
ODC	hsa-miR-320a/b	Bone marrow	HMSC	[127]
SAT1	hsa-miR-1-3p	Brain, cervix, kidney	Brain, HELA, HEK293T	[128] [121]
SAT1	hsa-miR-133a-3p	Kidney	HELA	[118]
SAT1	hsa-miR-210-3p	Stomach, gastric	AGS, MKN45	[129]
SAT1	hsa-miR-21-3p	Aortic	HAEC	[130]
SAT1	hsa-miR-34a-5p	Colon	HCT116	[131]
SAT1	hsa-miR-29b-3p	Mammary gland	BT474	[132]
SAT1	hsa-miR-9-3p	Brain	Brain	[128]
SMS	hsa-miR-181-5p	Kidney	HELA	[133]
SMS	hsa-miR-29b-3p	Pancreas	PBC	[119]
SMS	hsa-miR-1-3p	Kidney	HELA	[121]
SAT1	hsa-miR-132-3p	Pancreas	PBC	[119]
PAOX	hsa-miR-146a-5p	Peripheral blood	Jurkat	[134]
SAT1	hsa-miR-320c	Brain	Brain	[135•]
SMOX	hsa-miR-320c	Brain	Brain	[135•]
SMS	mmu-miR-1a-3p	Embryo	3T3	[121]
ODC	mmu-miR-21a-5p	Bone marrow	Macrophages	[121]
ODC	mmu-miR-1a-3p	Embryo	3T3	[121]
PAOX	mmu-miR-1a-3p	Embryo	3T3	[121]

**Fig. 2:**
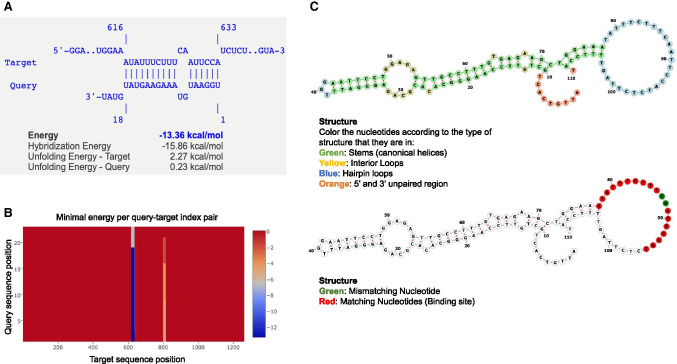
RNA-RNA binding prediction and interaction of hsa-miR-1-3p and ODC. RNA-RNA binding prediction was performed using the IntaRNA online tool (http://rna.informatik.uni-freiburg.de) to measure the hybridization energy between ODC1 (NM_001287189.2) and the hsa-miR-1-3P (MI0000651). The binding prediction of the microRNA and the target mRNA sequence revealed a hybridization energy of − 15.86 kcal/mol in exon 2 position 77 to position 94 with two mismatching nucleotides at position 87 and 88 (**A**, **B**). A secondary structure analysis was performed using Forna (Kerpedjiev P, Hammer S, Hofacker IL, 2015). Forna (force-directed RNA): Simple and effective online RNA secondary structure diagrams. Bioinformatics 31(20):3377–9.) server in Vienna’s RNA lab tool to reveal the binding position of the microRNA in the target sequence. The top structure of **C** represents color-coded mRNA secondary structure of ODC1 Exon 2. The nucleotide colors represent the type of structure they form, green: stems (canonical helices), red: multiloops (junctions), yellow: interior loops, blue: hairpin loops, orange: 5′ and 3′ unpaired region. The bottom structure represents the binding site of the microRNA, the green color represents mismatching Nucleotide, while the red color represents matching nucleotides at the binding site

**Fig. 3:**
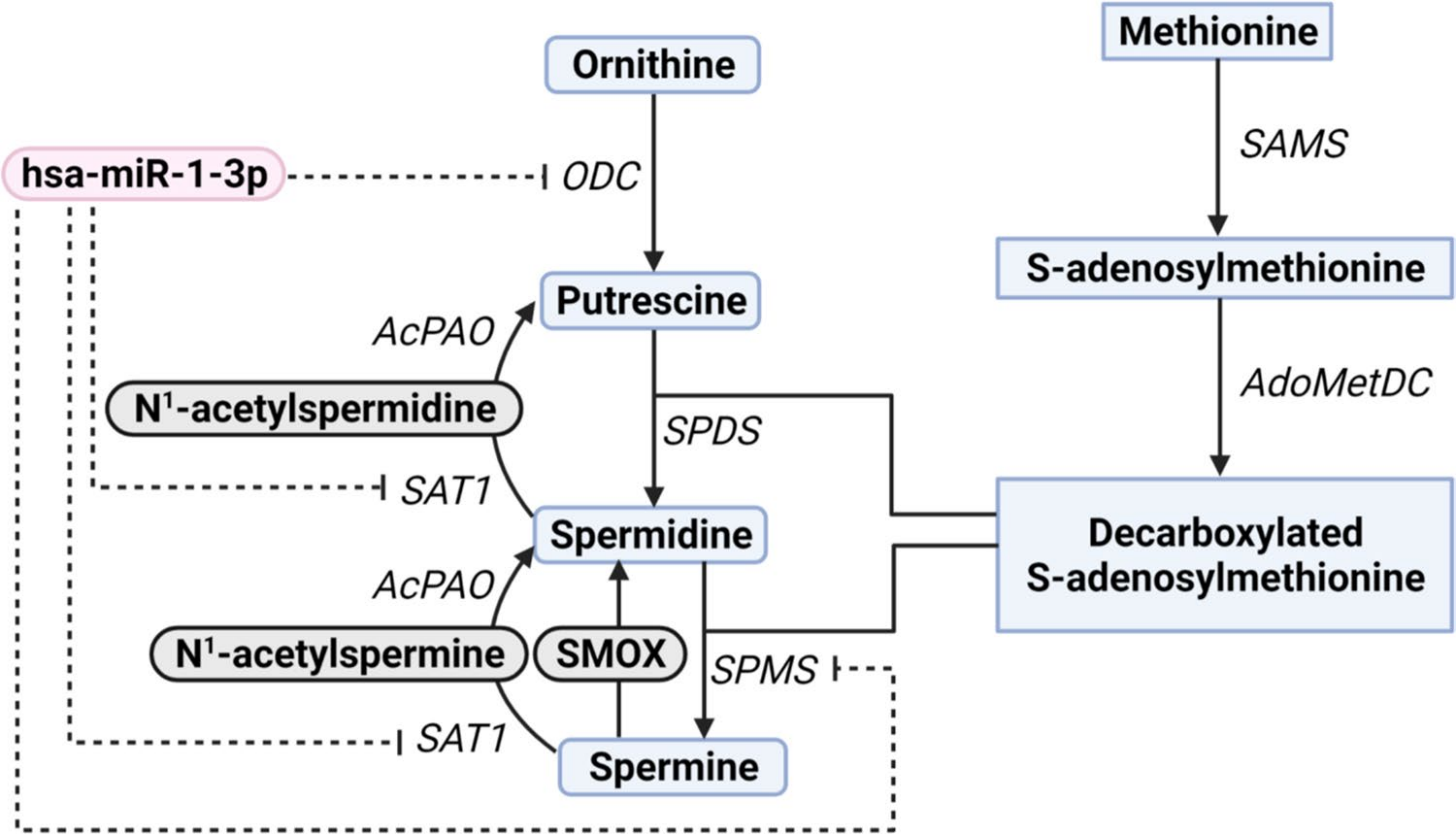
Gene interactions of hsa-miR-1-3p within the polyamine biosynthesis pathway. This figure shows hsa-miR-1-3p’s known interactions in the polyamine biosynthesis pathway from microarray and co-immunoprecipitation experiments collected from the Tarbase v8 database. This data analysis resulted in hsa-miR-1-3p 3′ UTR binding association with Ornithine Decarboxylase (ODC), Spermine synthase (SPMS), and Spermidine/spermine N1-acetyltransferase (SAT1). Other major polyamine biosynthesis enzymes and metabolites depicted are spermidine synthase (SPDS), acetylated polyamine oxidase (AcPAO), S-adenosylmethionine synthase (SAMS), S-adenosylmethionine decarboxylase (AdoMetDC), and Spermine Oxidase (SMOX)

Utilizing RNA binding prediction software, it is possible to examine the feasibility of cardiac miRNA targets within the polyamine biosynthesis pathway in silico, such as the predicted binding between miR-1 and ODC (Fig. 2). miR-1 is overexpressed and ODC activity is drastically decreased in the DM heart [69•, 87]. These findings along with in silico prediction results suggest a potential role of the dysregulated miRNA-polyamine axis in DMCM (Fig. 3).

## Summary and Future Direction

In this review, we summarized potential roles of polyamines in the progression of DMCM and the regulatory roles of miRNA in maintaining the synthesis and metabolism of polyamines. We have also provided a list of putative candidate miRNAs that are involved in the regulation of cardiac polyamines in DMCM. As DM is a metabolic disease, we elaborated the specific roles of polyamines in cardiac metabolism. In the DM heart, fat metabolism is increased, and mitochondrial function is impaired. Thus, we have elaborated how polyamines are involved in the regulation of fatty acid beta-oxidation and mitochondrial respiration. We have also elaborated how polyamines are involved in ketogenesis.

Despite intensive glycemic control, the risk of heart failure is high in DM patients [88•]. Thus, there is no cure for DM-induced heart failure, and a novel therapeutic approach is warranted. Regulation of cardiac polyamines could prove to be a potential novel avenue to ameliorate DMCM and prevent DM-induced heart failure. MiRNAs and their predicted targets described in this article warrant empirical studies to determine their specific roles in polyamine biosynthesis and cardiac metabolism in DMCM.

## Supplementary Information

Below is the link to the electronic supplementary material.

Supplementary file1 (PDF 395 KB)
