# Supplementary material for: Regulating Polyamine Metabolism by miRNAs in Diabetic Cardiomyopathy
Source: Curr Diab Rep. 2021 Dec 13;21(12):52. doi: 10.1007/s11892-021-01429-w (PMC8668854; doi:10.1007/s11892-021-01429-w)
Supplement: Supplementary file 1 — Supplementary file1 (PDF 395 KB) [file 11892_2021_1429_MOESM1_ESM.pdf]

COLLEGE OF MEDICINE
Department of Cellular and Integrative Physiology

September 17, 2021

To
Jose C. Florez, MD, PhD
Editor-in-Chief
Current Diabetes Report

Dear Dr. Florez

On behalf of all co-authors, I would like to thank you, section Editor Dr. Vanita Aroda, and Managing Editor Danielle Adams for your decision on our manuscript (appended below).

Per recommendation, we have revised our manuscript and included responses to comments below. I have also attached publication license from BioRender for Figures 1 and 3.

I hope our revised manuscript is acceptable for publication. Once again, thank for giving us opportunity to submit our work in your esteem journal.

Sincerely
Paras
____

Paras Kumar Mishra, Ph.D.
Fellow of American Heart Association
Fellow of American Physiological Society Cardiovascular Section
President, Midlands Society of Physiological Sciences
Director, Cardiopulmonary Function in Health and Disease
Chair, A Ross McIntyre Cardiorenal Seminar
Consulting Editor, AJP- Heart and Circulatory Physiology
Associate Editor, Frontiers in Integrative Physiology
Associate Professor
Department of Cellular & Integrative Physiology,
University of Nebraska Medical Center
601 S. Saddle Creek Road
DRC-1 Room 5047, Omaha, NE, 68198-5850
Phone: 1-402-559-8524
Fax: 1-402-559-4438
Email:paraskumar.mishra@unmc.edu
Website: https://www.unmc.edu/physiology/faculty/mishra.html
Twitter: @DrParasKMishra

Decision letter

Date: Sep 13, 2021
To: "Paras Kumar Mishra" paraskumar.mishra@unmc.edu
From: "Current Diabetes Reports" cyrillekate.castillo@springernature.com
Subject: Decision on your Manuscript #DBTS-D-21-00123
Dear Dr. Mishra:
Thank you very much for submitting your manuscript entitled 'Regulating polyamines metabolism by miRNAs in diabetic
cardiomyopathy' to Current Diabetes Reports.
I have decided that your manuscript could be accepted for publication should you be prepared to incorporate minor revisions.
When preparing your revised manuscript, you are asked to carefully consider the comments below and submit a list of
responses to the comments.
Your list of responses should be uploaded as a file in addition to your revised manuscript.

In order to submit your revised manuscript electronically, please access the following web site:
https://www.editorialmanager.com/dbts/

Your username is: ********
If you forgot your password, you can click the 'Send Login Details' link on the EM Login page.
Click 'Author Login' to submit your revision.
We look forward to receiving your revised manuscript.
Sincerely yours,
Danielle Adams
Managing Editor
Current Diabetes Reports
COMMENTS FOR THE AUTHOR:
****
Before this is sent to Dr. Aroda for review, please revise the following formatting elements:
1. Abstract: Should be structured with the following 3 headings: Purpose of Review, Recent Findings, Summary
2. Bulleted/Annotated References: Choose 2-3 recent references to mark as "important" (1 bullet) or "very important" (2
bullets). Under each chosen reference, write 1 or 2 sentences to explain to readers why it stands out.
**Authors may need to take specific actions to achieve compliance with funder and institutional open access
mandates.** If your research is supported by a funder that requires immediate open access (e.g. according to Plan S
principles) then you should select the gold OA route, and we will direct you to the compliant route where possible. For authors selecting the subscription publication route our standard licensing terms will need to be accepted, including our self-archiving policies. Those standard

licensing terms will supersede any other terms that the author or any third party may assert apply to any version of the manuscript.

Find out more about compliance
Our flexible approach during the COVID-19 pandemic
If you need more time at any stage of the peer-review process, please do let us know. While our systems will continue to
remind you of the original timelines, we aim to be as flexible as possible during the current pandemic.
This letter contains confidential information, is for your own use, and should not be forwarded to third parties.

Response to comments

Comment 1: Abstract: Should be structured with the following 3 headings: Purpose of Review, Recent Findings, Summary

Response: In the revised manuscript, we have formatted Abstract accordingly.

Comment 2: Bulleted/Annotated References: Choose 2-3 recent references to mark as "important" (1 bullet) or "very important" (2 bullets). Under each chosen reference, write 1 or 2 sentences to explain to readers why it stands out.

Response: References are formatted accordingly in the revised manuscript.

49 Spadina Ave. Suite 200
Toronto ON M5V 2J1 Canada
www.biorender.com

Confirmation of Publication and Licensing Rights

September 16th, 2021
Science Suite Inc.

Subscription:	*Institution*
Agreement number:	*EU22YPUITO*
Journal name:	*Current Diabetes Reports*

To whom this may concern,

This document is to confirm that **Paras Kumar Mishra** has been granted a license to use the BioRender content, including icons, templates and other original artwork, appearing in the attached completed graphic pursuant to BioRender's Academic License Terms. This license permits BioRender content to be sublicensed for use in journal publications.

All rights and ownership of BioRender content are reserved by BioRender. All completed graphics must be accompanied by the following citation: “Created with BioRender.com”.

BioRender content included in the completed graphic is not licensed for any commercial uses beyond publication in a journal. For any commercial use of this figure, users may, if allowed, recreate it in BioRender under an Industry BioRender Plan.

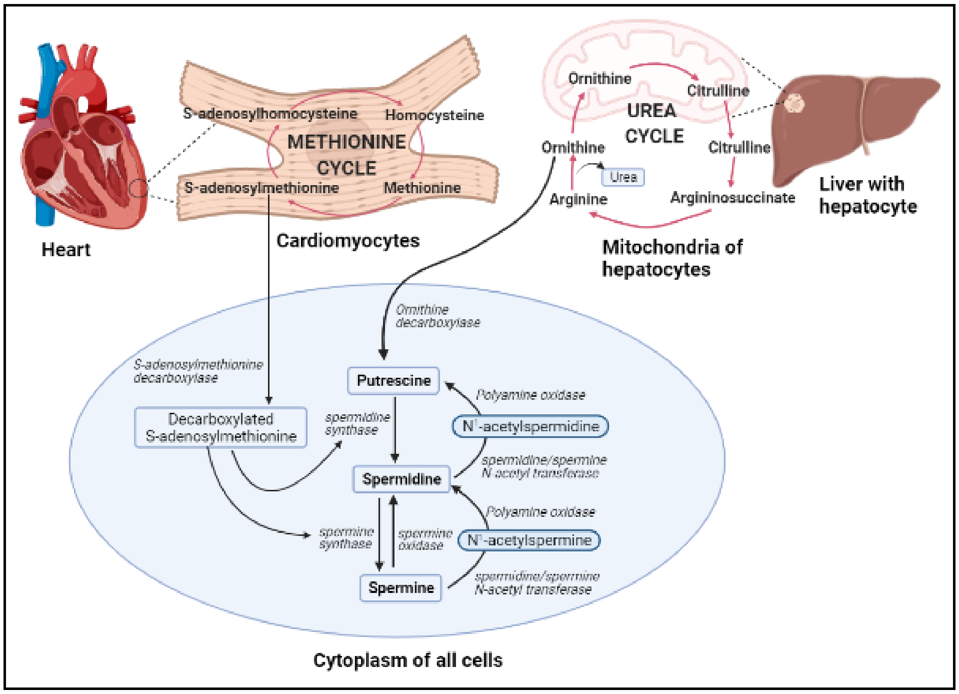

For any questions regarding this document, or other questions about publishing with BioRender refer to our BioRender Publication Guide, or contact BioRender Support at support@biorender.com.

49 Spadina Ave. Suite 200
Toronto ON M5V 2J1 Canada
www.biorender.com

Confirmation of Publication and Licensing Rights

September 16th, 2021
Science Suite Inc.

Subscription:	*Institution*
Agreement number:	*WV22YPUQ2N*
Journal name:	*Current Diabetes Reports*

To whom this may concern,

This document is to confirm that **Paras Kumar Mishra** has been granted a license to use the BioRender content, including icons, templates and other original artwork, appearing in the attached completed graphic pursuant to BioRender's Academic License Terms. This license permits BioRender content to be sublicensed for use in journal publications.

All rights and ownership of BioRender content are reserved by BioRender. All completed graphics must be accompanied by the following citation: "Created with BioRender.com".

BioRender content included in the completed graphic is not licensed for any commercial uses beyond publication in a journal. For any commercial use of this figure, users may, if allowed, recreate it in BioRender under an Industry BioRender Plan.

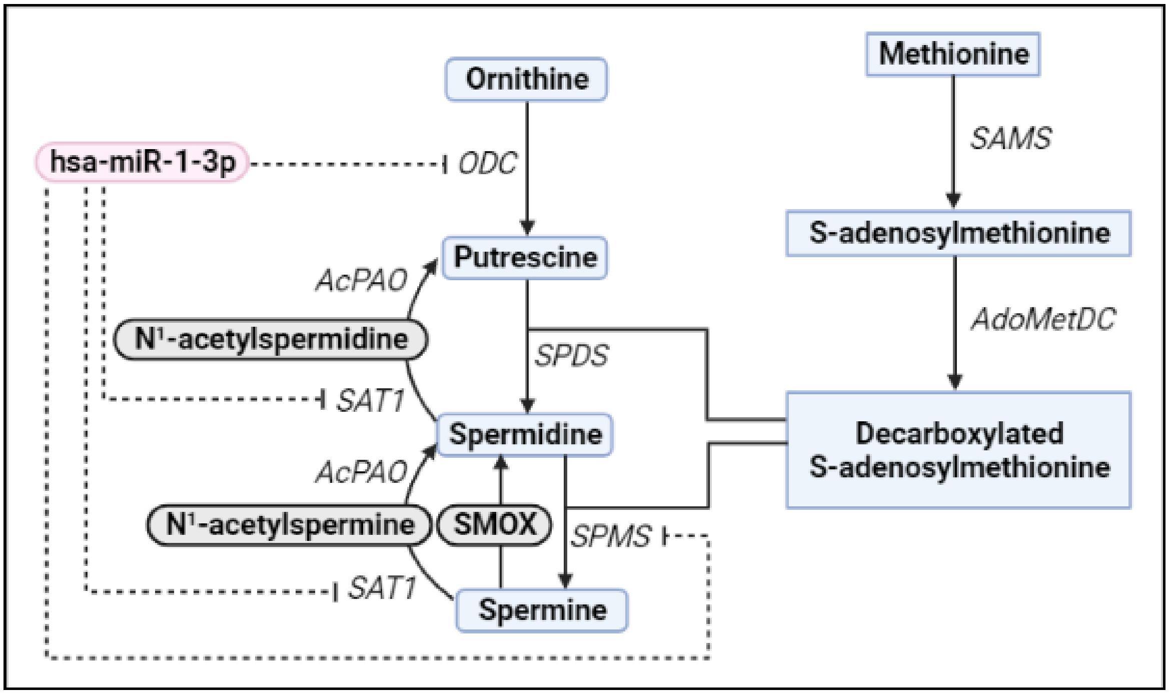

For any questions regarding this document, or other questions about publishing with BioRender refer to our BioRender Publication Guide, or contact BioRender Support at support@biorender.com.
